# Comparison between three different equations for the estimation of glomerular filtration rate in predicting mortality after coronary artery bypass

**DOI:** 10.1186/s12882-019-1564-y

**Published:** 2019-10-16

**Authors:** Sandro Gelsomino, Massimo Bonacchi, Fabiana Lucà, Fabio Barili, Stefano Del Pace, Orlando Parise, Daniel M. Johnson, Michele Massimo Gulizia

**Affiliations:** 1 Cardiothoracic Department, Maastricht University Hospital, Florence, Italy; 2 Department of Cardiothoracic Surgery, Cardiovascular Research Institute Maastricht University, Universiteitssingel 50, 6229 ER Maastricht, The Netherlands; 3 Cardiothoracovascular Department, Careggi University Hospital, Florence, Italy; 4 ANMCO Research Center of Heart Care, Florence, Italy; 5 Department of Cardiovascular Surgery, S. Croce Hospital, Cuneo, Italy; 6 Cardiology Garibaldi-Nesima Hospital, Catania, Italy

**Keywords:** Coronary artery bypass, Renal function, Glomerular filtration, Risk score

## Abstract

**Background:**

This study was undertaken to compare the accuracy of chronic kidney disease-epidemiology collaboration (eGFR_CKD-EPI_) to modification of diet in renal disease (eGFR_MDRD_) and the Cockcroft-Gault formulas of Creatinine clearance (C_CG_) equations in predicting post coronary artery bypass grafting (CABG) mortality.

**Methods:**

Data from 4408 patients who underwent isolated CABG over a 11-year period were retrieved from one institutional database. Discriminatory power was assessed using the c-index and comparison between the scores’ performance was performed with DeLong, bootstrap, and Venkatraman methods. Calibration was evaluated with calibration curves and associated statistics.

**Results:**

The discriminatory power was higher in eGFR_CKD-EPI_ than eGFR_MDRD_ and C_CG_ (Area under Curve [AUC]:0.77, 0.55 and 0.52, respectively). Furthermore, eGFR_CKD-EPI_ performed worse in patients with an eGFR ≤29 ml/min/1.73m^2^ (AUC: 0.53) while it was not influenced by higher eGFRs, age, and body size. In contrast, the MDRD equation was accurate only in women (calibration statistics *p* = 0.72), elderly patients (*p* = 0.53) and subjects with severe impairment of renal function (*p* = 0.06) whereas C_CG_ was not significantly biased only in patients between 40 and 59 years (*p* = 0.6) and with eGFR 45–59 ml/min/1.73m^2^ (*p* = 0.32) or ≥ 60 ml/min/1.73m^2^ (*p* = 0.48).

**Conclusions:**

In general, CKD-EPI gives the best prediction of death after CABG with unsatisfactory accuracy and calibration only in patients with severe kidney disease. In contrast, the CG and MDRD equations were inaccurate in a clinically significant proportion of patients.

## Background

Preoperative renal impairment is a well-established predictor of adverse outcomes in patients undergoing coronary artery bypass grafting (CABG) [1–3]. In addition, with advances in the fields of nephrology, cardiology, and cardiac surgery, an increasing number of patients with renal dysfunction are being offered coronary revascularization [4].Therefore, accurate preoperative evaluation of renal function is recommended before CABG [5].

Estimated glomerular filtration rate (eGFR) is now considered a more sensitive marker of renal function than serum creatinine alone identifying patients with even mild renal impairment despite normal or nearly normal creatinine levels [6–8].

Different formulas to estimate eGFR have been implemented [1, 2, 9] and, amongst these, the Modification of Diet in Renal Disease (MDRD) equation (eGFR_MDRD_) [1, 2, 9], the more recently defined Chronic Kidney Disease-Epidemiology Collaboration (CKD_EPI_) equation (eGFR_CKD-EPI_) [10, 11]along with the Cockcroft-Gault equation formula of Creatinine Clearance (C_CG_)[7, 8, 12]are the most widely employed.

The predictive value of eGFR on mortality and morbidity following CABG has been widely demonstrated [9, 10, 11, 12]. Nonetheless, papers have concentrated on patients with serum creatinine or eGFR calculated by the C_CG_ equation or MDRD [2, 13, 14] and, at the best of our knowledge, no study exists comparing eGFR_MDRD_ and C_CG._ eGFR_CKD-EPI_ in their predictive value of post-CABG mortality.

Therefore, in this study we test the reliability of these three formulae in predicting mortality after CABG and compare their discrimination and calibration power. In addition, discrimination and calibration of the three models were also evaluated in relation to factors that may influence the absolute bias of the equations [15].

## Methods

This study was performed in accordance with the Declaration of Helsinki and following STROBE guidelines [16]. Consecutive patients undergoing isolated CABG at Careggi Hospital (Florence, Italy) between 2006 and 2017 were retrospectively enrolled in the study.

### Definitions

Definitions and calculations were as in our previous research [17]. Kidney dysfunction was defined following the recently updated Kidney Disease Outcomes Quality Initiative (KDOQI) [18] and Kidney Disease Improving Global Outcomes (KDIGO) Guidelines [7].

The C_CG_ [19], MDRD [20] and Chronic Kidney Disease (CKD)-EPI estimate of renal function were calculated as recommended [15, 21] and normalized to 1.73 m^2^ of the body surface area (BSA) [22] and expressed in ml/min/1.73m^2^. The body mass index (BMI) was calculated as body weight divided by the square of height, with body weight expressed in kg and height in meters.

### Endpoint

The single endpoint was all-cause mortality within 30 days after CABG (*n* = 3880 cases, 79 deaths) or during index procedure hospitalization- in case of postoperative length of stay > 30 days (*n* = 528 cases, 36 deaths) which was reported via hospital records or registry information.

### Statistical analysis

Continuous data were summarized as mean and standard deviation or median and twenty-fifth to seventy-fifth percentiles in case of skewed distributions. Frequencies were reported for categorical variables. The performances of C_CG_ vs. eGFR_CKD-EPI_ vs. eGFR_MDRD_ were analyzed to determine their discrimination power and calibration [23, 24]. The discrimination performance was assessed by receiver operating characteristic (ROC) and the area under the curve (AUC) with 95% confidence intervals [25–27]. Curves were analyzed with De Long, bootstrap, and Venkatraman methods [27]. Furthermore, the model was tested by Somers’ test assuming predictions as perfectly discriminating when D_xy_ = 1 [28]. Moreover, we employed the Brier score and when it was equal to 0 the prediction could be considered perfect [29].

The calibration performance can be evaluated by generating calibration plots: the perfect calibrated predictions stay on the diagonal, whilst a curve below or above it, respectively, reflects overestimation and underestimation [23, 27, 30].

Agreement between observed frequency and predicted probabilities were tested with the Hosmer-Lemeshow (H-L) goodness-of-fit test, whereas the comparison of actual slope and intercept with the ideal value of 1 and 0 was performed with the U statistic and tested against a χ^2^ distribution with 2 degrees of freedom.

Discrimination and calibration performances were stratified by renal function, gender, age, body weight, and BMI due to the fact that these variables might influence the performance of the equations. Stratification of calculated eGFR (≥ 60 ml/min/1.73m^2^;45–59 ml/min/1.73m^2^, 30–44 ml/min/1.73m^2^ and ≤ 29 ml/min/1.73m^2^) was based on updated KDOQI and KDIGO [7, 19] and according to level of calculated EGFR, as well as on the basis of the estimates of the Cockcroft- Gault, MDRD, and CKD-EPI formulas. Using Cohen’s k we tested the agreement between calculated and estimated EGFR .

Clinical cutoffs were used for age (18 to 39, 40 to 59, and ≥ 60 years) [31] and body weight (≤59, 60 to 79, 80 to 99, and ≥ 100 kg) [12]. Stratification for BMI followed the World Health Organization guidelines [32]. To address missing values (Additional file 1), we used fully conditional specification [33] (FCS) multiple imputation (MI) method (1000 replications).

R, version 3.3.1 (R Foundation for Statistical Computing, Wien, Austria) with pROC, rms and Resource Selection packages was employed to carry out statistical analysis.

Significance for hypothesis testing was set at the 0.05 two-tailed level.

## Results

### Study population

After exclusion of subjects without an available plasma creatinine level (*n* = 86), body weight (*n* = 73) or height (*n* = 37) measurements, those undergoing preoperative dialysis (*n* = 18), who had undergone previous cardiac surgery (*n* = 108), who experienced significant (life-threatening) post-operative complications (*n* = 396) or with mitral insufficiency ≥ moderate (*n* = 174) the final population consisted of 4408 subjects who remained eligible for inclusion. Patient characteristics are presented in Table 1.

**Table 1:** Patient characteristics (*n* = 4408)

Baseline Characteristics	
Age	70.7 [64.5–76.2]
Female sex	902 (20.5)
BSA	1.85 ± 0.17
BMI	26.2 ± 3.8
Diabetes	793 (17.9)
COPD	154 (3.5)
PVD	525 (11.9)
CVD	26 (0.6)
MI < 30 days	873 (19.8)
Unstable Angina	1494 (33.9)
≥ 3 vessels disease	2579 (58.5)
LVEF	48.5 [42.1–57.6]
Creatinine (mg/dL)	1.09 [0.91–1.31]
EuroScore	8.9 [4.5–12.2]
STS Score	9.9 [4.8–13.1]
STS PROM	9.2 [4.2–12.8]
eGFR_MDRD_	
≥ 60	2494 (56.6)
45–59	1200 (27.2)
30–44	638 (14.5)
≤ 29	76 (1.7)
eGFR_CKD-EPI_	
≥ 60	2597 (58.9)
45–59	1097 (24.9)
30–44	633 (14.4)
≤ 29	81 (1.8)
C_CG_	
≥ 60	2440 (55.4)
45–59	1170 (26.5)
30–44	703 (15.9)
≤ 29	95 (2.2)
Operative Characteristics	
1 graft	264 (6.0)
2 grafts	1943 (44.1)
≥ 3 grafts	2201 (49.9)
OPCAB	1983 (44.9)

Data are shown as mean ± SD or numbers (percentage) or median [Interquartile range]. Abbreviations. *BSA*: Body Surface Area; *BMI*: Body Mass Index; *COPD*: Chronic Obstructive Pulmonary Disease; *PVD*: Peripheral Vascular Disease; *CVD*: Cerebrovascular Disease; *MI*: Myocardial Infarction; *LVEF*: Left Ventricular Ejection Fraction; *STS*: Society of Thoracic Surgeons; *PROM*: Predicted risk of mortality; *eGFRMDRD*: Glomerular Filtration Rate estimated by the Modification in Diet in Renal Disease; *eGFRCKD-EPI*: (ml/min/1.73 m2) Glomerular Filtration Rate estimated by Chronic Kidney Disease-Epidemiology Collaboration equation (ml/min/1.73 m2); *CCG*: Creatinine Clearance estimated by Cockroft-Gault formula (ml/min/1.73 m2); *OPCAB*: Off-Pump Coronary Artery Bypass

### Mortality

Overall early mortality was 2.6% (*n* = 115): it was 24/2440 (1%) in patients with EGFR ≥60 ml/min/1.73m^2^, 20/1170 (1.7%) in those with EGFR between 45 and 59 ml/min/1.73m^2^, 39/703 (5.5%) in subjects with EGFR ranging from 30 to 44 ml/min/1.73m^2^ and 32/95 (33.6%) in those with EGFR ≤29 ml/min/1.73m^2^.

### Overall performance

**Fig. 1:**
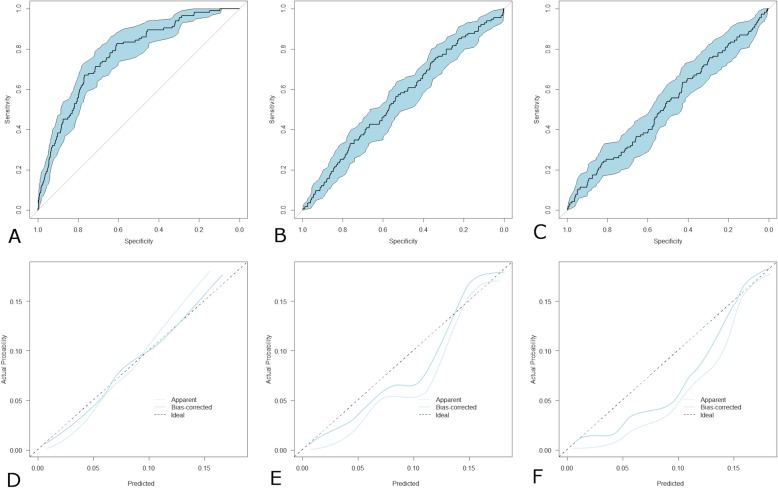
Receiver operating characteristic curves with 95% confidence intervals for eGFR_CKD-EPI_ (**a**) eGFR_MDRD_ (**b**) and C_CG_ (**c**). A curve lying on the diagonal line reflects the performance of a diagnostic test that is no better than chance level. The closer is the curve to the upper left-hand corner the greater is the discriminant testing capacity. Calibration plots of eGFR_CKD-EPI_ (**d**) and eGFR_MDRD_ (**e**) and C_CG_ (**f**). The diagonal line represents the perfect calibration. If the line lies below the ideal curve, the EGFR formula overestimates the outcome, if it is above the ideal curve the formula underestimates the outcome

Results of Predictive Performance, Discrimination Power and Calibration are shown in Tables 2 and 3. The c Statistic and the other measures of performance showed that only the CKD-EPI formula had any notable discriminatory power. The MDRD formula shows borderline significant discrimination, given the lower confidence limit for the C statistic is 0.50 The CG formula shows no evidence of being able to discriminate between those who died and those who did not. The ROC curves are plotted in Fig. 1 a-c: The AUC was higher in eGFR_CKD-EPI_ than in the other two and all the comparisons amongst them showed significant differences between the three formulas with best performance by eGFR_CKD-EPI_.

**Table 2:** Predictive performance and discrimination power

		eGFR	AUC	95% CI	De Long	Bootstrap	Venkatraman	Somers	Brier
All		CKD-EPI	0.77	0.73–0.81	< 0.01^*^	< 0.01^*^	< 0.01^*^	0.54	0.01
		MDRD	0.55	0.50–0.61	0.65^†^	0.61^†^	0.76^†^	0.11	0.02
		CG	0.52	0.47–0.57	< 0.01^‡^	< 0.01^‡^	< 0.01^‡^	0.04	0.02
eGFR	> = 60 ml/min./1.73m^2^	CKD-EPI	0.78	0.73–0.82	< 0.01^*^	< 0.01^*^	< 0.01^*^	0.55	0.01
		MDRD	0.57	0.50–0.63	< 0.01^†^	< 0.01^†^	< 0.01^†^	0.13	0.02
		CG	0.73	0.69–0.75	0.57^‡^	0.54^‡^	0.60^‡^	0.44	0.01
	45–59 ml/min./1.73m^2^	CKD-EPI	0.67	0.57–0.77	< 0.01^*^	< 0.01^*^	< 0.01^*^	0.34	0.01
		MDRD	0.59	0.49–0.69	< 0.01^†^	< 0.01^†^	< 0.01^†^	0.18	0.02
		CG	0.65	0.60–0.69	0.67^‡^	0.66^‡^	0.69^‡^	0.30	0.01
	30–44 ml/min./1.73m^2^	CKD-EPI	0.63	0.55–0.71	0.04^*^	0.04^*^	0.04^*^	0.25	0.03
		MDRD	0.53	0.40–0.67	0.45^†^	0.43^†^	0.55^†^	0.07	0.05
		CG	0.57	0.43–0.71	0.04^‡^	0.04^‡^	0.04^‡^	0.15	0.04
	<=29 ml/min./1.73m^2^	CKD-EPI	0.53	0.35–0.71	0.35^*^	0.32^*^	0.54^*^	0.06	0.13
		MDRD	0.65	0.48–0.82	0.10^†^	0.09^†^	0.16^†^	0.30	0.14
		CG	0.49	0.27–0.71	0.23^‡^	0.20^‡^	0.28^‡^	0.02	0.13
Age	AGE > =60 y	CKD-EPI	0.68	0.63–0.74	< 0.01^*^	< 0.01^*^	< 0.01^*^	0.37	0.02
		MDRD	0.57	0.51–0.63	0.58^†^	0.52^†^	0.63^†^	0.14	0.03
		CG	0.54	0.48–0.60	< 0.01^‡^	< 0.01^‡^	< 0.01^‡^	0.09	0.03
	AGE 40–59 y	CKD-EPI	0.81	0.70–0.92	< 0.01^*^	< 0.01^*^	< 0.01^*^	0.62	0.02
		MDRD	0.59	0.48–0.70	< 0.01^†^	< 0.01^†^	< 0.01^†^	0.17	0.03
		CG	0.86	0.80–0.91	0.45^‡^	0.41^‡^	0.52^‡^	0.72	0.01
	AGE 18–39 y	CKD-EPI	0.82	0.72–0.96	< 0.01^*^	< 0.01^*^	< 0.01^*^	0.64	0.06
		MDRD	0.57	0.46–0.72	0.03^†^	0.02^†^	0.04^†^	0.14	0.09
		CG	0.47	0.08–0.85	< 0.01^‡^	< 0.01^‡^	< 0.01^‡^	0.06	0.12
Gender	Male	CKD-EPI	0.76	0.71–0.82	< 0.01^*^	< 0.01^*^	< 0.01^*^	0.53	0.01
		MDRD	0.55	0.49–0.61	0.04^†^	0.04^†^	0.04^†^	0.10	0.02
		CG	0.51	0.45–0.57	< 0.01^‡^	< 0.01^‡^	< 0.01^‡^	0.03	0.02
	Female	CKD-EPI	0.72	0.66–0.78	< 0.01^*^	< 0.01^*^	< 0.01^*^	0.44	0.02
		MDRD	0.55	0.43–0.66	0.03^†^	0.03^†^	0.04^†^	0.09	0.04
		CG	0.50	0.43–0.57	< 0.01^‡^	< 0.01^‡^	< 0.01^‡^	0.01	0.06
Weight	> = 100 Kg	CKD-EPI	0.81	0.66–0.97	0.01^*^	0.01^*^	0.02^*^	0.63	0.05
		MDRD	0.50	0.31–0.70	0.04^†^	0.04^†^	0.04^†^	0.01	0.07
		CG	0.48	0.26–0.69	< 0.01^‡^	< 0.01^‡^	< 0.01^‡^	0.04	0.08
	80–99 Kg	CKD-EPI	0.77	0.69–0.84	< 0.01^*^	< 0.01^*^	< 0.01^*^	0.53	0.01
		MDRD	0.52	0.41–0.62	0.04^†^	0.04^†^	0.04^†^	0.04	0.02
		CG	0.50	0.40–0.62	< 0.01^‡^	< 0.01^‡^	< 0.01^‡^	0.03	0.03
	60–79 Kg	CKD-EPI	0.79	0.74–0.84	< 0.01^*^	< 0.01^*^	< 0.01^*^	0.59	0.01
		MDRD	0.56	0.49–0.63	0.02^†^	0.02^†^	0.03^†^	0.12	0.02
		CG	0.50	0.43–0.57	< 0.01^‡^	< 0.01^‡^	< 0.01^‡^	0.01	0.03
	<=59 Kg	CKD-EPI	0.69	0.56–0.83	0.01^*^	0.03^*^	0.02^*^	0.39	0.03
		MDRD	0.55	0.41–0.69	0.48^†^	0.45^†^	0.56^†^	0.11	0.03
		CG	0.53	0.38–0.68	< 0.01^‡^	< 0.01^‡^	< 0.01^‡^	0.07	0.04
BMI	> = 30 Kg/m^2^	CKD-EPI	0.81	0.71–0.92	< 0.01^*^	< 0.01^*^	< 0.01^*^	0.63	0.01
		MDRD	0.53	0.37–0.69	0.03^†^	0.03^†^	0.04^†^	0.06	0.02
		CG	0.46	0.28–0.64	< 0.01^‡^	< 0.01^‡^	< 0.01^‡^	0.06	0.03
	25–29 Kg/m^2^	CKD-EPI	0.76	0.70–0.82	< 0.01^*^	< 0.01^*^	< 0.01^*^	0.52	0.01
		MDRD	0.55	0.47–0.63	0.01^†^	< 0.01^†^	0.02^†^	0.10	0.02
		CG	0.45	0.37–0.53	< 0.01^‡^	< 0.01^‡^	< 0.01^‡^	0.01	0.04
	18.5–24 Kg/m^2^	CKD-EPI	0.77	0.70–0.83	< 0.01^*^	< 0.01^*^	< 0.01^*^	0.53	0.01
		MDRD	0.57	0.49–0.66	0.00^†^	< 0.01^†^	0.01^†^	0.15	0.02
		CG	0.47	0.39–0.56	< 0.01^‡^	< 0.01^‡^	< 0.01^‡^	0.04	0.04
	<=18.5 Kg/m^2^	CKD-EPI	0.77	0.62–0.92	0.04^*^	0.04^*^	0.04^*^	0.54	0.14
		MDRD	0.52	0.27–0.66	0.65^†^	0.62^†^	0.70^†^	0.03	0.13
		CG	0.53	0.13–0.94	< 0.01^‡^	< 0.01^‡^	< 0.01^‡^	0.07	0.12

Best performance for: Brier score = 0, AUC = 1, Somers’ Dxy = 1. Abbreviations: *eGFR*: estimated Glomerular Filtration Rate; *CI*: Confidence Interval; *CKD-EPI*: Chronic Kidney Disease-Epidemiology Collaboration Formula; MDRD: Modification of Diet in Renal Disease Formula; CG: Cockroft-Gault Formula; BMI: Body Mass Index.* CKD-EPI vs MDRD; † MDRD vs CG; ‡ CG vs CKD-EPI

**Table 3:** Calibration

		eGFR	Slope	Intercept	U statistic	Hosmer-Lemeshow
All		CKD-EPI	0.69	0.12	0.38	0.40
		MDRD	1.34	−1.06	0.03	0.04
		CG	1.29	−1.00	0.02	0.02
eGFR	> = 60 ml/min/1.73m^2^	CKD-EPI	0.60	0.15	0.21	0.58
		MDRD	1.20	−1.16	0.03	0.02
		CG	0.57	0.18	0.39	0.48
	45–59 ml/min/1.73m^2^	CKD-EPI	0.54	−0.14	0.15	0.78
		MDRD	1.17	1.56	0.02	0.03
		CG	0.59	0.20	0.28	0.32
	30–44 ml/min/1.73m^2^	CKD-EPI	0.49	0.18	0.09	0.39
		MDRD	0.22	−0.16	0.02	0.04
		CG	0.28	−0.17	0.03	0.03
	<=29 ml/min/1.73m^2^	CKD-EPI	1.13	0.58	0.04	0.04
		MDRD	1.07	−0.36	0.07	0.06
		CG	1.23	−0.28	0.04	0.03
Age	AGE > =60 y	CKD-EPI	1.03	0.30	0.10	0.69
		MDRD	0.91	−0.04	0.43	0.53
		CG	0.63	−1.35	0.02	0.03
	AGE 40–59 y	CKD-EPI	0.66	−0.24	0.25	0.41
		MDRD	0.60	−1.77	0.02	0.03
		CG	0.92	−0.03	0.56	0.60
	AGE 18–39 y	CKD-EPI	0.75	−0.88	0.32	0.37
		MDRD	0.71	−1.75	0.02	0.03
		CG	0.59	−1.65	0.01	0.02
Gender	Male	CKD-EPI	0.85	0.36	0.20	0.10
		MDRD	0.70	−1.67	0.03	0.03
		CG	0.84	−1.43	0.02	0.03
	Female	CKD-EPI	0.65	−0.30	0.30	0.19
		MDRD	0.81	0.89	0.44	0.72
		CG	0.79	−1.33	0.03	0.03
Weight	> = 100 Kg	CKD-EPI	0.77	−0.28	0.39	0.49
		MDRD	0.30	−1.43	0.01	0.02
		CG	0.50	−1.30	0.02	0.02
	80–99 Kg	CKD-EPI	0.65	−0.16	0.40	0.64
		MDRD	0.20	−1.72	0.02	0.04
		CG	0.46	−1.65	0.01	0.02
	60–79 Kg	CKD-EPI	0.56	0.13	0.19	0.43
		MDRD	0.70	0.11	0.31	0.59
		CG	0.72	−1.47	0.02	0.02
	<=59 Kg	CKD-EPI	0.63	0.18	0.10	0.32
		MDRD	0.70	0.12	0.39	0.80
		CG	0.88	0.20	0.40	0.47
BMI	> = 30 Kg/m^2^	CKD-EPI	0.87	0.13	0.58	0.67
		MDRD	0.50	−2.07	0.01	0.04
		CG	0.47	−1.68	0.01	0.03
	25–29 Kg/m^2^	CKD-EPI	0.75	−0.39	0.50	0.57
		MDRD	0.40	−2.05	0.01	0.03
		CG	0.40	−1.75	0.02	0.02
	18.5–24 Kg/m^2^	CKD-EPI	0.65	−0.26	0.42	0.86
		MDRD	0.70	0.23	0.50	0.70
		CG	0.46	−1.42	0.01	0.02
	<=18.5 Kg/m^2^	CKD-EPI	0.85	0.71	0.16	0.36
		MDRD	0.80	−0.44	0.48	0.73
		CG	0.79	−0.37	0.37	0.50

Best performance for: Slope = 1, Intercept = 0, non-significant *P*-values of the U statistic, and Hosmer–Lemeshow test. Abbreviations: *eGFR*: estimated Glomerular Filtration Rate; *CI*: Confidence Interval; *CKD-EPI*: Chronic Kidney Disease-Epidemiology Collaboration Formula; MDRD: Modification of Diet in Renal Disease Formula; *BMI*: Body Mass Index; *CG*: Cockroft-Gault Formula

The pattern of calibration (Fig. 1 d-f) was different between the three indices. Indeed, eGFR_CKD-EPI_ was closer to the ideal line with a slight under-prediction when risk was higher but with non-significant *p* values for the calibration statistics (both, *p* = 0.40). In contrast, eGFR_MDRD_ and C_CG_ diverged significantly from the ideal diagonal with significant p values for the related summary statistics (both, *p* = 0.02).

### Performance by kidney function

**Fig. 2:**
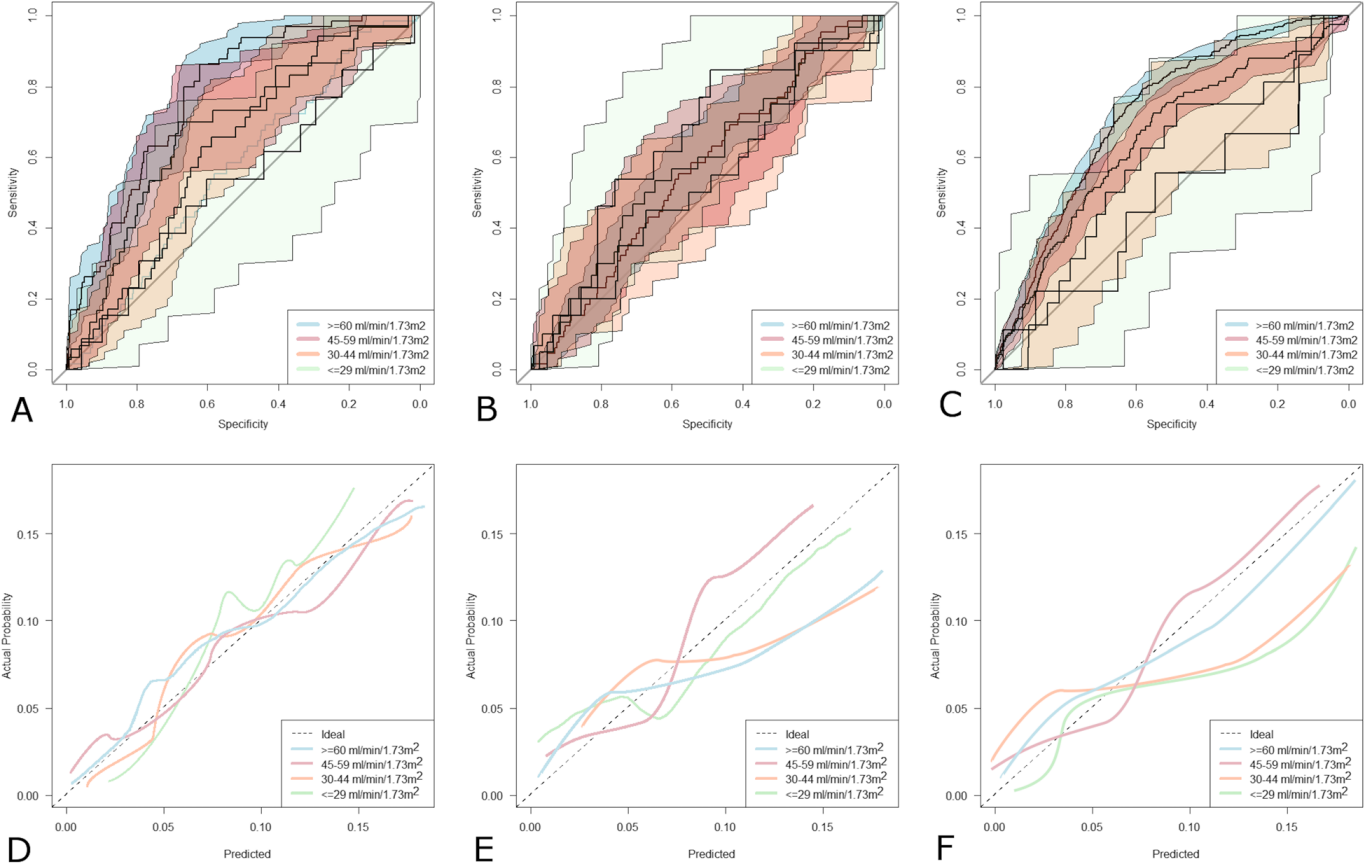
Patients Stratified by Renal Function. Receiver operating characteristic curves with 95% confidence intervals for eGFR_CKD-EPI_ (**a**) eGFR_MDRD_ (**b**). and C_CG_ (**c**) . Colored curve above the diagonal line perform progressively better the closer they are to the upper left-hand corner. Calibration plots of eGFR_CKD-EPI_ (**d**) eGFR_MDRD_ (**e**) and C_CG_ (**f**). Lines below the ideal curve (dotted line) overestimate the outcome, if they lie above the ideal curve the outcome is underestimated

Patients with a higher eGFR were younger (− 0.13 years [95%CI 0.11–1.1] per ml/min/1.73m^2^ increment in eGFR) with a higher body weight (1.64 kg [95%CI 1.41–2.89]) and a higher BMI (0.62 kg/m^2^ [95%CI 0.48–0.96]). The analysis of discrimination in the subgroups demonstrated an overall worse performance of eGFR_MDRD_, (Fig. 2a-c) with significant differences when eGFR was > 29 ml/min/1.73m^2^. In contrast, when the eGFR was ≤29 ml/min/1.73m^2^ the performance of MDRD was superior (*p* = 0.14). C_CG_ showed comparable performance of eGFR_CKD-EPI_ when eGFR was > 44 ml/min/1.73m^2^.

The pattern of calibration was different in the different subgroups of patients (Fig. 2 e-f: eGFR_CKD-EPI_ demonstrated a satisfactory calibration with eGFR > 29 ml/min/1.73m^2^ but with non-significant *p* values for the calibration statistics (*p* = 0.58, *p* = 0.78, *p* = 0.39, in the three groups with eGFR > 29 ml/min/1.73m^2,^ respectively). However, it tended to under- influence of predict mortality when eGFR was ≤29 ml/min/1.73m^2^ (*p* = 0.04).

In contrast, eGFR_MDRD_ was well calibrated at values of eGFR ≤29 ml/min/1.73m^2^ (*p* = 0.06) whereas it diverged significantly from perfect calibration when eGFR was higher than 29 ml/min/1.73m^2^ (*p* = 0.02, *p* = 0.03, *p* = 0.04, in the three groups with eGFR > 29 ml/min/1.73m^2,^ respectively). Finally, C_CG_ tended to over-prediction when eGFR was < 44 ml/min/1.73m^2^ (*p* = 0.03).

### Performance by age

**Fig. 3:**
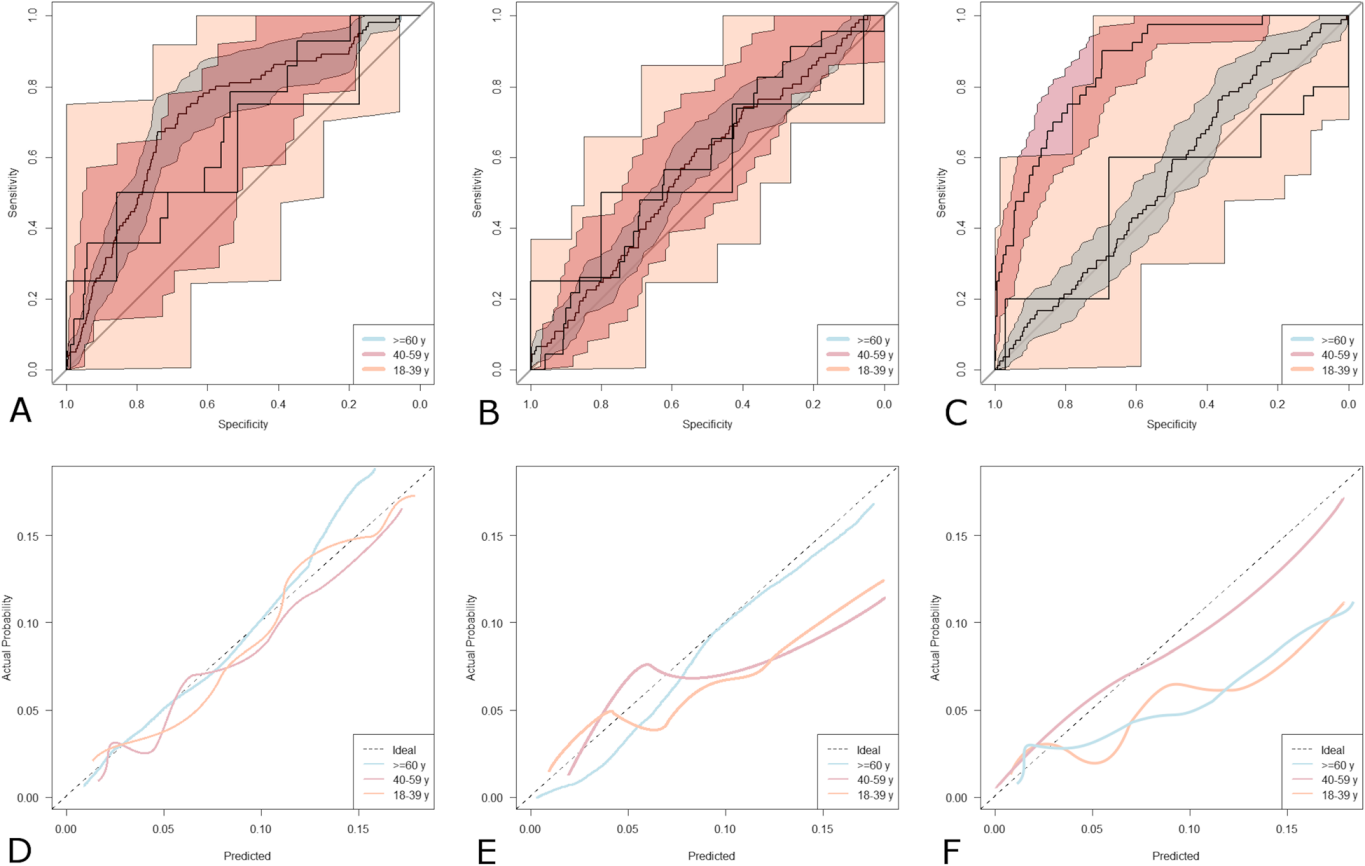
Patients Stratified by Age. Receiver operating characteristic curves with 95% confidence intervals for eGFR_CKD-EPI_ (**a**) eGFR_MDRD_ (**b**) and C_CG_ (**c**) stratified by age. Colored curve above the diagonal line perform progressively better the closer they are to the upper left-hand corner. Calibration plots of eGFR_CKD-EPI_ (**d**) and eGFR_MDRD_ (**e**) and C_CG_ (**f**). Lines below the ideal curve (dotted line) overestimate the outcome, if they lie above the ideal curve the outcome is underestimated

Older patients had lower eGFR (− 0.93 ml/min/1.73m^2^ [95%CI 0.71–1.13] per yearly increment in age), had a lower body weight (− 1.73 kg [95%CI 1.51–2.29]) and a lower BMI (− 0.56 kg/m^2^ [95%CI 0.48–0.75]). The AUC of the ROC curves (Fig. 3 a-c) was significantly higher for eGFR_CKD-EPI_ in all subgroups. C_CG_ performed better the MDRD equation in the range of 40–59 years whereas it showed the worst performance of the three groups < 40 years.

The pattern of calibration was different amongst age subgroups (Fig. 3 d-f): eGFR_CKD-EPI_ was close to the ideal diagonal in the oldest patients whereas it tended to slightly overestimate in the other age groups with non-significant *p* values for the calibration statistics (*p* = 0.69). The eGFR_MDRD_ resulted to be well calibrated in the ≥60 year- subgroup (*p* = 0.53) whereas it demonstrated a significant tendency to over-estimation in the other age subgroups (all, *p* < 0.05). Finally, C_CG_ tended to over-prediction in the ≥60 year- and 18–39 year-subgroups (both, *p* = 0.03).

### Performance by gender

Compared with men, woman had a lower body weight (− 11.4 kg [95%CI 4.4–12.4]) and a lower BMI (− 1.65 kg/m^2^ [95%CI 1.14–6.65]).

**Fig. 4:**
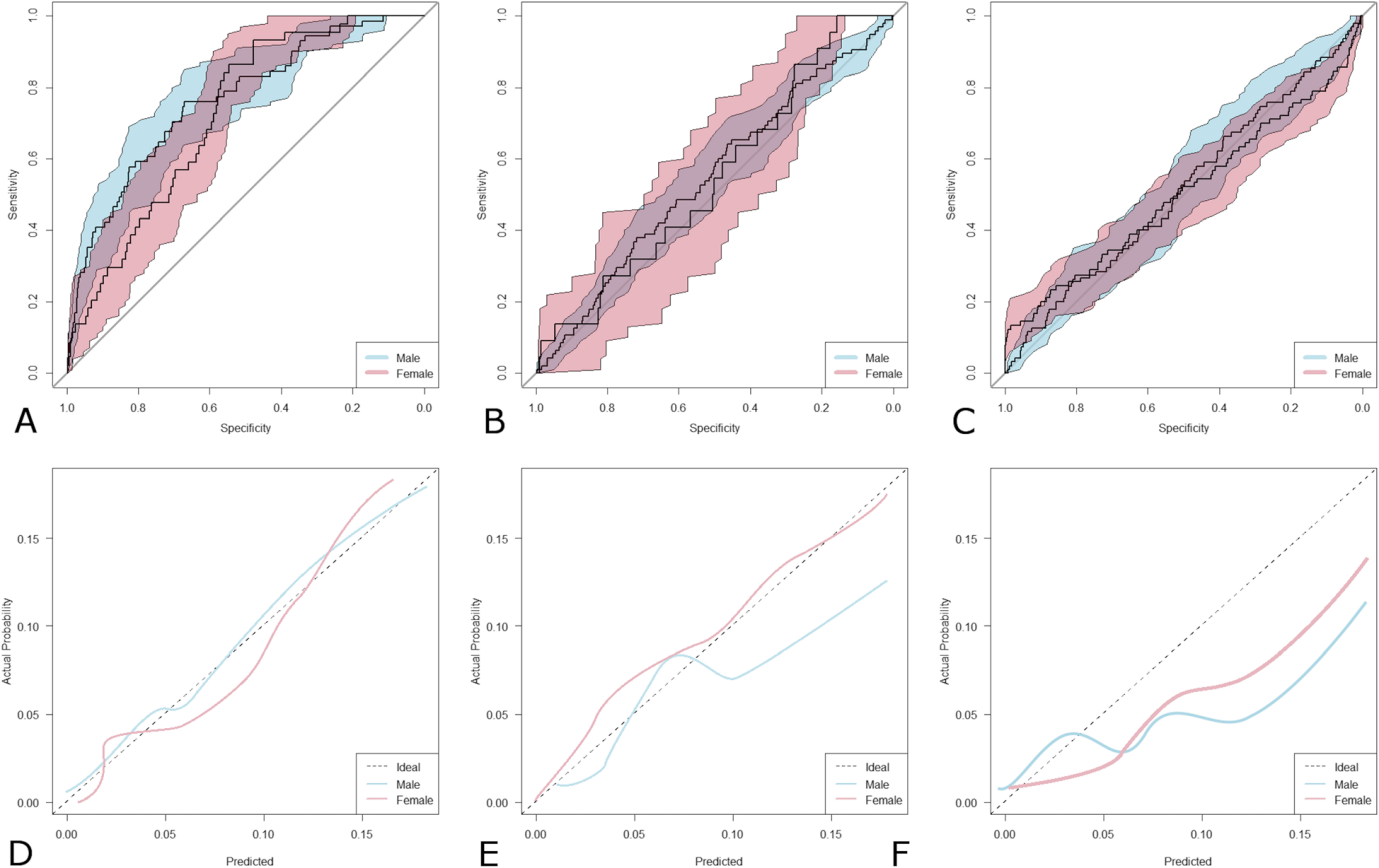
Patients Stratified by Gender. Receiver operating characteristic curves with 95% confidence intervals for eGFR_CKD-EPI_ (**a**) eGFR_MDRD_ (**b**) and C_CG_ (**c**). . Colored curve above the diagonal line perform progressively better the closer they are to the upper left-hand corner. Calibration plots of eGFR_CKD-EPI_ (**d**) eGFR_MDRD_ (**e**) and C_CG_ (**f**). Lines below the ideal curve (dotted line) overestimate the outcome, if they lie above the ideal curve the outcome is underestimated

The eGFR_CKD-EPI_ equation showed a higher AUC in both genders (Fig. 4 a-c) with significant differences compared to the C_CG_ and MDRD equations (*p* < 0.05 for all comparisons). C_CG_ showed a worse performance compared to eGFR_MDRD_ in both genders.

In men (Fig. 4 d-f) eGFR_CKD-EPI_ reached maximum accuracy whereas it showed a tendency to overestimation in women although calibration statistics were not significant in (both,*p* = 0.1). In contrast, eGFR_MDRD was_ accurate in women (*p* = 0.72) and tended to overestimation in men (*p* = 0.03) whereas C_CG_ significantly overestimated in both sexes (both, p = 0.03).

### Performance by BMI

Subjects with a higher BMI were younger (− 0.22 years [95%CI 0.09–0.44] each kg/m^2^), had a higher body weight (2.89 kg [95%CI 2.73–3.21] each kg/m^2^) and a higher eGFR (0.78 ml/min/1.73m^2^ [95%CI 0.56–0.91] each kg/m^2^).

**Fig. 5:**
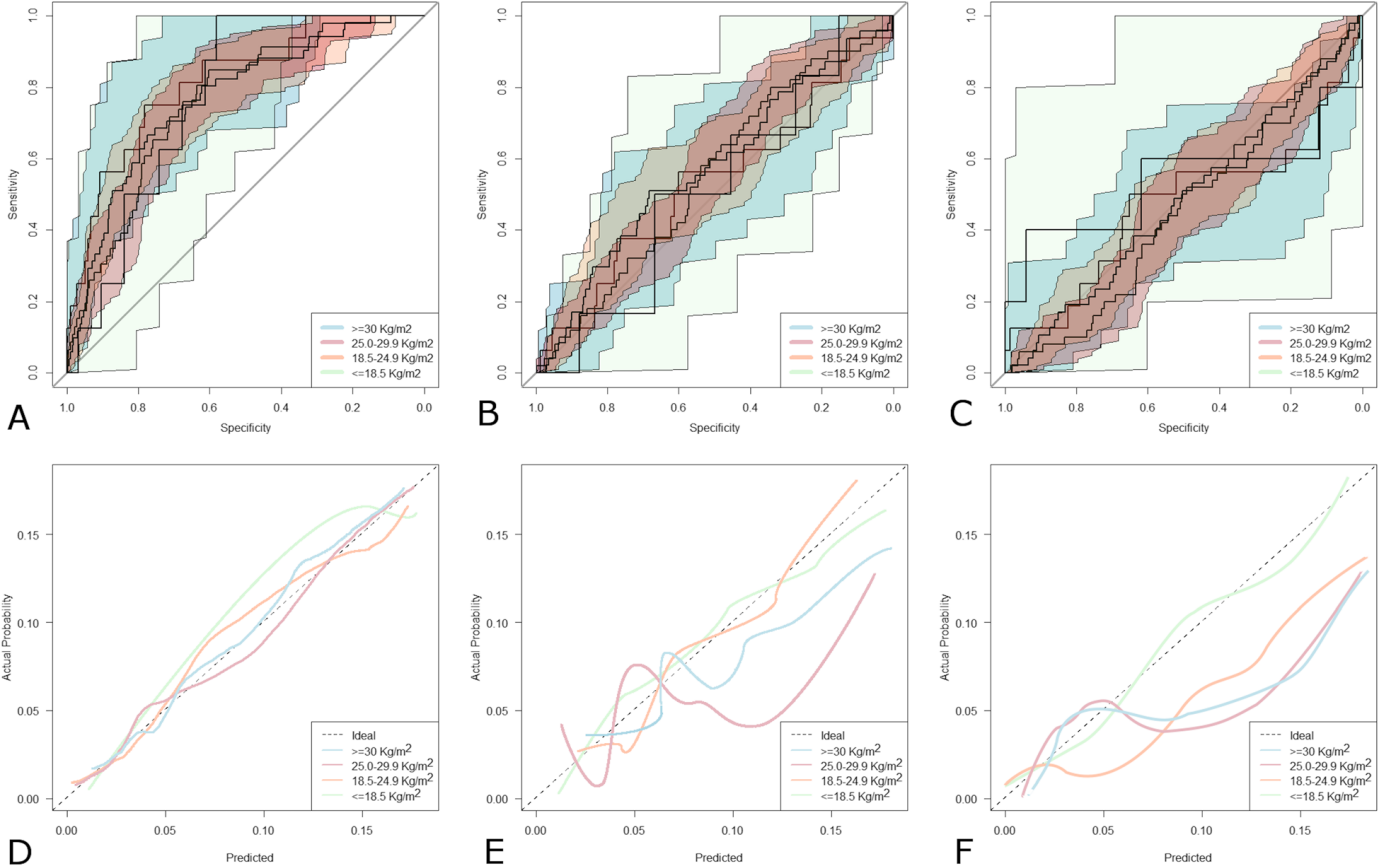
Patients Stratified by Body Mass Index (BMI). Receiver operating characteristic curves with 95% confidence intervals for eGFR_CKD-EPI_ (**a**) and eGFR_MDRD_ (**b**). and C_CG_ (**c**). . Colored curve above the diagonal line perform progressively better the closer they are to the upper left-hand corner. Calibration plots of eGFR_CKD-EPI_ (**d**) eGFR_MDRD_ (**e**) and C_CG_ (**f**). Lines below the ideal curve (dotted line) overestimate the outcome, if they lie above the ideal curve the outcome is underestimated

The AUC of ROC curves (Fig. 5 a-c) were higher with eGFR_CKD-EPI_ no matter what the BMI subgroup was (*p* < 0.05 for all comparisons). The calibration curves are shown in Fig. 5 D-F: the eGFR_CKD-EPI_ equation was close to the ideal diagonal at any value of BMI with a slight lower accuracy in patients with BMI < 25.0 kg/m^2^ (*p* = 0.03). In contrast, the MDRD was more accurate in patients with BMI < 25.0 kg/m^2^ (*p* = 0.7) whereas it showed a trend to over-prediction in subjects with BMI ≥25.0 kg/m^2^ (*p* = 0.03 and *p* = 0.04 in patients with BMI 25–29 Kg/m^2^ and ≥ 30 Kg/m^2^, respectively). Finally, the C_CG_ formula was the less accurate up to 18.5 kg/m^2^ (p = 0.03, *p* = 0.02 and p = 0.02 in patients with BMI > 30 Kg/m^2^, 25–29 Kg/m^2^ and ≥ 30 Kg/m^2^, respectively) with a tendency to over-prediction, while it was comparable to the MDRD formula when in patients with BMI < 25.0 kg/m^2^ (*p* = 0.5).

## Discussion

Patients with CAD and renal disease have a dismal prognosis [34, 35]. In addition, estimated glomerular filtration rate (eGFR) has a major impact on the outcome of patients undergoing coronary revascularization, either percutaneous coronary intervention or coronary artery bypass grafting (CABG) [10, 36] .

Reduced erythropoietin synthesis and consequent anemia and reduced 1,25(OH) vitamin D production, associated with increased parathyroid hormone levels and higher prevalence of vascular calcification and arteriosclerosis have been reported to explain the association between renal dysfunction and cardiovascular events [37, 38].

In addition, patients with reduced or impaired renal function face additional challenges in the setting of CABG for several reasons: 1) Concomitant factors such as including advanced age, low ejection fraction, history myocardial infarction, and stroke which are themselves determinants of poor outcomes [39]. 2) Detrimental cardiovascular effects by oxidative stress and high levels of homocysteine, hyperuricemia, hypercalcemia, and uremia associated with reduced renal function [40, 41]. 3) Higher incidence of multivessel disease and microvessel disease in such patients [2, 11].

However, little is known whether eGFRs calculated with different formulas have comparable predictive value on post-CABG mortality.

In our recent paper [42] we had shown that the eGFR_CKD-EPI_ equation led to categorization with a significantly lower number of patients at risk for post- CABG complications and with cut-off values of eGFR_CKD-EPI_ predicting early and late events significantly lower than accepted prediction threshold values for post- CABG unfavorable events [2, 41, 43].

In the present study our study we assessed the performance, in terms of discrimination and calibration, of the MDRD, CG formulas and CKD-EPI equations in predicting mortality after CABG in the whole patient population and across different subgroups of patients defined by eGFR, age, gender and body size.

The main findings of our study can be summarized as follows:

1. The overall performance of eGFR_CKD-EPI_ in prediction of post-CABG death is significantly superior to both eGFR_MDRD_ and CG formulas and its calibration curve is close to the ideal prediction over a wide range of thresholds for mortality risk prediction whereas the MDRD and CG equations, show a general trend towards over-prediction.
2. The CKD-EPI equation gave the best overall accuracy and agreement after classification in subgroups of GFR. Furthermore, it had a greater accuracy in patients with an eGFR > 30 ml/min/1.73m^2^ whereas it showed a trend towards under-predicting mortality when the eGFR fell below 30 ml/min/1.73m^2^. In contrast, eGFR_MDRD_ confirmed [15] to be the most reliable in patients with highly compromised renal function whilst C_CG_ showed comparable performance of eGFR_CKD-EPI_ when eGFR was > 44 ml/min/1.73m^2^.
3. Previous studies have demonstrated that the performance of eGFR equations depends on the stage of CKD [44], thus being greatly influenced by the value of glomerular filtration rate [15]. In addition, the MDRD equation resulted in imprecise and underestimates of eGFR at higher renal function levels [45]. In our experience, the accurateness of the CKD- EPI formula in predicting post-CABG mortality was independent of age and gender whereas eGFR_MDRD_ overestimated the prediction in younger patients and in men while it was accurate in women and patients ≥60 years and C_CG_ tended to over-prediction in the ≥60 year- and 18–39 year-subgroups and in both genders. This might be related to the uncertain reliability of these formulas in reflecting the true renal function [46, 47]
4. Since all three formulas rely on serum creatinine as the indicator for the rate of glomerular filtration and because serum creatinine correlates with muscle mass and nutritional status, the performance of the formulas might be influenced by body composition. This was assessed by studying the influence of body mass or BMI on eGFR, which, in our experience, did not affect the CKD-EPI equation whose calibration curve was close to the ideal diagonal at any value of BMI. In contrast, the MDRD was accurate only in overweight patients and those with body mass ≥ 30.0 kg/m2. These results are in accordance with Michels et al. [15] who found that MDRD provided greatest accuracy in defining renal function (97.0%) in subjects with the highest body weight whereas other studies showed no relation or positive correlation concluding that no creatinine-based method is reliable in the obese [48]. Lastly, the CCG formula was the least accurate up to 18.5 kg/m2 while it was comparable to the MDRD formula in smaller patients.

Renal function is regularly included in all risk stratification models in cardiac surgical patients. Two well-recognized risk models assess cardiovascular outcomes of patients undergoing CABG: the EuroSCORE and the Society of Thoracic Surgeons (STS) National Adult Cardiac Database [49]. The first employs eGFR calculated with C_CG_ formula and value ranges that are not concordant with National Kidney Foundation recommendations [50] whereas the STS risk score incorporates a continuous parameter for serum creatinine and a binary variable for hemodialysis [51]. Based on KDIGO clinical practice Guidelines [8] and previous evidence [15], it would be of great interest to test, in a broad patient population, the eGFR_CKD-EPI_ formula incorporated into CABG risk prediction algorithm, re-estimating the weight for all the variables in the predictive tool, to compare the predictive performance of such a model to algorithms currently in use. At this point, in the absence of validation studies, it is impossible to understand whether the use of eGFR_CKD-EPI in_ stratification models would make a valuable contribution to improve the predictive value of the algorithm. Further research is warranted.

### Study limitations

This study has some limitation that should be highlighted. Firstly, its retrospective nature makes it impossible to draw final conclusions. Secondly, the population is relatively small, and assessment of the equations was carried out in a restricted study population (i.e. post CABG patients), limiting extrapolation of findings to other cohorts such as myocardial infarction, heart failure etc. Thirdly, the patient population has several variations from most CABG profiles: low number of female, low incidence of adult onset diabetes mellitus, unstable angina and MI < 30 days and high number of patients receiving 1–2 grafts. Fourthly, patients with associated procedures were excluded and this could introduce another bias. We wanted to test the three indices excluding as much as possible confounding factors. Fifthly, preoperative eGFR was calculated on a single measurement and therefore susceptible of being influenced by cardiac function and therapy. Sixthly, preoperative renal function was unknown which could have post-CABG survival. Seventhly, eGFR_CKD-EPI_ still has the limitation of being related to muscle mass, thus other filtration markers such as serum cystatin might have helped us in overcoming this issue. Eighthly, data presented in this paper did not say anything about which equation is the better predictor of true GFR, but it was beyond the aim of the paper that was explore which eGFR formula is the best predictor of mortality. The two things may go hand-in-hand, but this cannot be concluded from the existing data and it will be object of upcoming research. Finally, neither we compare the performance of the three formulae within specific risk scores, nor did not test the performance of eGFR_CKD-EPI_ on postoperative renal failure but these were beyond the aim of the present study.

## Conclusions

In general, CKD-EPI gives the best prediction of death after CABG with unsatisfactory accuracy and calibration only in patients with severe CKD. In contrast, the CG and MDRD equations were inaccurate in predicting mortality in a clinically significant proportion of patients. eGFR_CKD-EPI_ should be incorporated into CABG risk-assessment algorithms to provide patients and their family members the most accurate risk prediction.

## Supplementary information

**Additional file 1.** Missing Data. Frequencies of missing data.

## Data Availability

The datasets used and/or analysed during the current study are available from Orlando Parise on reasonable request.

## Abbreviations

AUC: Area under curve
BMI: Body mass index
BSA: Body surface area
CABG: Coronary artery bypass grafting
CCG: Creatinine Clearance estimated by Cockroft-Gault formula
CKD: Chronic kidney disease AUC: Area under curve
eGFR_CKD-EPI_: Glomerular Filtration Rate estimated by Chronic Kidney Disease-Epidemiology Collaboration equation
eGFR_MDRD_: Glomerular Filtration Rate estimated by the Modification in Diet in Renal Disease
FCS: Fully conditional specification
H-L: Hosmer-Lemeshow goodness-of-fit test
KDIGO: Kidney Disease Improving Global Outcomes
KDOQI: Kidney Disease Outcomes Quality Initiative
MI: Multiple imputation
OPCAB: Off-Pump Coronary Artery Bypass
ROC: Receiver operating characteristic
