# Supplementary material for: Comparison between three different equations for the estimation of glomerular filtration rate in predicting mortality after coronary artery bypass
Source: BMC Nephrol. 2019 Oct 16;20:371. doi: 10.1186/s12882-019-1564-y (PMC6796478; doi:10.1186/s12882-019-1564-y)
Supplement: Supplementary file 1 — Additional file 1. Missing Data. Frequencies of missing data. [file 12882_2019_1564_MOESM1_ESM.docx]

| **Missing Data** |  |
| --- | --- |
|  | (%) |
|  |  |
| Age | 0.000 |
| Sex | 0.000 |
| BSA | 0.000 |
| BMI | 0.000 |
| Diabetes | 0.002 |
| COPD | 0.001 |
| PVD | 0.011 |
| CVD | 0.003 |
| MI< 30days | 0.005 |
| LVEF | 0.006 |
| Creatinine | 0.000 |
| EGFR_MDRD_ | 0.000 |
| EGFR_CKD-EPI_ | 0.000 |
| C_CG_ | 0.000 |
| Number of Grafts | 0.006 |
| Graft details | 0.010 |
| CABG on-off pump | 0.004 |
|  |  |

**Abbreviations.** BSA: Body Surface Area; BMI: Body Mass Index; COPD: Chronic Obstructive Pulmonary Disease; PVD: Peripheral Vascular Disease; CVD: Cerebrovascular Disease; MI: Myocardial Infarction; LVEF: Left Ventricular Ejection Fraction; eGFR_MDRD_: Glomerular Filtration Rate estimated by the Modification in Diet in Renal Disease; eGFR_CKD-EPI_: Glomerular Filtration Rate estimated by Chronic Kidney Disease-Epidemiology Collaboration equation; C_CG_: Glomerular Filtration Rate estimated by Cockroft-Gault; CABG: Coronary artery bypass grafting.
